# Secukinumab attenuates reactive astrogliosis via IL‐17RA/(C/EBPβ)/SIRT1 pathway in a rat model of germinal matrix hemorrhage

**DOI:** 10.1111/cns.13144

**Published:** 2019-04-24

**Authors:** Sheng‐Peng Liu, Lei Huang, Jerry Flores, Yan Ding, Peng Li, Jun Peng, Gang Zuo, John H. Zhang, Jun Lu, Ji‐Ping Tang

**Affiliations:** ^1^ Department of Pediatrics, Affiliated Haikou Hospital of Xiangya Medical College Central South University Haikou China; ^2^ Department of Physiology and Pharmacology, School of Medicine Loma Linda University Loma Linda California; ^3^ Department of Neurosurgery, School of Medicine Loma Linda University Loma Linda California; ^4^ Department of Anesthesiology, School of Medicine Loma Linda University Loma Linda California

**Keywords:** IL‐17A, neurobehavior, reactive astrogliosis, secukinumab

## Abstract

**Aims:**

Reactive astrogliosis plays a critical role in neurological deficits after germinal matrix hemorrhage (GMH). It has been reported that interleukin‐17A and IL‐17A receptor IL‐17RA/(C/EBPβ)/SIRT1 signaling pathway enhances reactive astrogliosis after brain injuries. We evaluated the effects of secukinumab on reactive astrogliosis in a rat pup model of GMH.

**Methods:**

A total of 146 Sprague Dawley P7 rat pups were used. GMH was induced by intraparenchymal injection of collagenase. Secukinumab was administered intranasally 1 hour post‐GMH. C/EBPβ CRISPR or SIRT1 antagonist EX527 was administrated intracerebroventricularly (icv) 48 hours and 1 hour before GMH induction, respectively. Neurobehavior, Western blot, histology, and immunohistochemistry were used to assess treatment regiments in the short term and long term.

**Results:**

The endogenous IL‐17A, IL‐17RA, C/EBPβ, and GFAP and proliferation marker CyclinD1 were increased, while SIRT1 expression was decreased after GMH. Secukinumab treatment improved neurological deficits, reduced ventriculomegaly, and increased cortical thickness. Additionally, treatment increased SIRT1 expression and lowered proliferation proteins PCNA and CyclinD1 as well as GFAP expression. C/EBPβ CRISPR activation plasmid and EX527 reversed the antireactive astrogliosis effects of secukinumab.

**Conclusion:**

Secukinumab attenuated reactive astrogliosis and reduced neurological deficits after GMH, partly by regulating IL‐17RA/(C/EBPβ)/SIRT1 pathways. Secukinumab may provide a promising therapeutic strategy for GMH patients.

## 1 INTRODUCTION

Germinal matrix hemorrhage (GMH) is one of the leading causes of mortality and morbidity in preterm and low‐birthweight infants, occurring in approximately 3.5 per 1000 births in the United States.^1^, ^2^ The germinal matrix contains various thin‐walled blood vessel, which leaves them susceptible to spontaneous rupture due to respiratory and hemodynamic fluctuations seen in preterm infants.^3^, ^4^, ^5^ Currently, there is no effective therapeutics in the management of GMH,^6^ and new treatment strategies are warranted.

Although previous studies have shown that reactive astrogliosis (also known as astrocyte activation) is necessary for poststroke CNS repair,^7^, ^8^, ^9^ recent studies have demonstrated that reactive astrogliosis plays a pivotal role in neurological injury in CNS injury models such as hemorrhagic stroke.^10^, ^11^, ^12^, ^13^ Reactive astrogliosis is characterized by astrocyte hypertrophy and astrocyte proliferation, which have been shown to contribute to glial scar formation.^14^ Despite glial scarring being necessary to seal the site of injury and protect damaged neural tissue, this repair mechanism inhibited the regrowth of damaged axons which contributed to further neurological deficits brought on by CNS injury.^15^, ^16^

Interleukin‐17A and its receptor IL‐17R play a significant role in inflammation and BBB breakdown after stroke.^17^ IL‐17 is predominantly produced by various immune cells such as natural Th17 cells, γδT cells, T‐helper cells, and innate lymphoid cells.^18^ After hemorrhagic stroke, there is an increased level of IL‐17A, one of six subtypes of the IL‐17 family (IL‐17A to IL‐17F) within the CNS.^19^ IL‐17 receptor (IL‐17R) has five subtypes which include IL‐17RA to IL‐17RE, where IL‐17A and IL‐17F can bind to IL‐17RA. IL‐17RA is found on various cell types in the CNS such as microglia, neurons, and astrocytes.^20^, ^21^, ^22^ The CCAAT/enhancer binding proteins β (C/EBPβ), a transcription factor, has been reported to be activated by IL‐17RA/ACT1/TRAF6 signaling pathway.^17^, ^23^ Increased C/EBPβ expression has been shown to suppress the expression and activity of silent information regulator 1 (SIRT1).^24^, ^25^, ^26^ Previous studies have demonstrated that SIRT1 plays an essential role in cell proliferation and cell survival.^27^ SIRT1 attenuates reactive astrogliosis by targeting nuclear factor ƙB (NF‐ƙB)^28^, ^29^ and signal transducing activator of transcription 3 (STAT3).^30^ Thus, IL‐17A activation of the IL‐17RA/C/EBPβ signaling pathway may play a role in reactive astrogliosis, whereas SIRT1 signaling pathways attenuate reactive astrogliosis after GMH.

Secukinumab, a recombinant monoclonal antibody, selectively targets IL‐17A, is currently used as a treatment for severe chronic immune diseases that are associated with IL‐17A.^31^, ^32^ In this present study, we hypothesized that the inhibition of IL‐17A, via secukinumab, would attenuate C/EBPβ inhibitory effects on SIRT1 resulting in the reactive astrogliosis, thereby improving short‐ and long‐term neurological deficits after GMH in rats.

## 2 MATERIALS AND METHODS

### 2.1 Animals and GMH model

All procedures were approved by the Institutional Animal Care and Use Committee at Loma Linda University, and in accordance with the National Institute of Health guidelines for the treatment of animals.

Timed pregnant Sprague Dawley (SD) rats were purchased from Envigo (Livermore). The rat pups were housed with dams until sacrificed (short‐term time points) or weaned (long‐term time points); after the weaning process, each cage housed two rats of the same gender. All rats were housed in a controlled humidity and temperature room with a 12‐h light/dark cycle and were raised with free access to water and food. A total of 146 P7 rat pups (brain development comparable to 30‐32 weeks of human gestation) were used. GMH was induced by stereotactic‐guided injection of bacterial collagenase as previously described.^33^ Rat pups were fixed on a stereotaxic plate while anesthetized with 2%‐3% isoflurane (mixed with oxygen gas and air). A 10‐μL Hamilton syringe (Hamilton Co.) was used to inject 0.3 units of collagenase VII‐S (Sigma) at stereotactic coordinates from bregma of 1.6 mm (right lateral), 1.5 mm (rostral) and 2.7 mm (depth) from the dura. After injection, the needle remained for 5 minutes and then was withdrawn slowly for an additional 5 minutes to minimize potential “back‐leakage.” The burr hole created for the insertion of the Hamilton syringe was sealed with bone wax and the incision site sutured. Respiration, heartbeat, skin color, myodynamia, and body temperature were monitored before and after recovery from anesthesia. After recovery, rat pups were placed back with the dam. Sham animal groups were subjected to needle insertion without collagenase injection.^33^

### 2.2 Animal experimental groups and treatments

**Figure 1:**
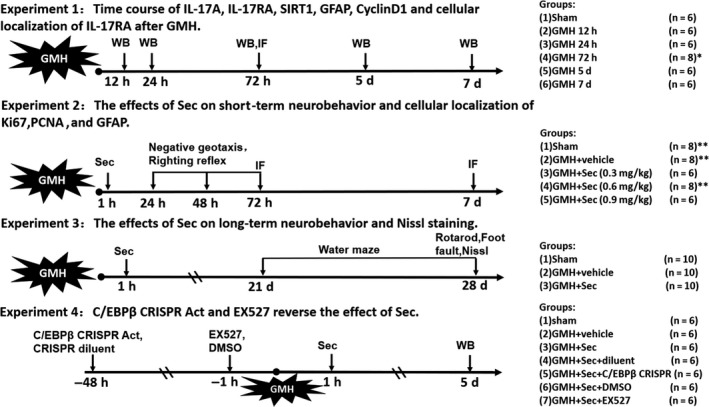
Experimental design and animal groups. C/EBPβ, CCAAT/enhancer binding protein beta; CRISPR, clustered regularly interspaced short palindromic repeats; DMSO, dimethyl sulfoxide; GFAP, glial fibrillary acidic protein; GMH, germinal matrix hemorrhage; IF, immunofluorescence; IL‐17RA, interleukin‐17 receptor; PCNA, proliferating cell nuclear antigen; Sec, secukinumab; WB, Western blot. ^*^Extra 2 pups were used for IF (IL‐17RA and GFAP) at 72 h after GMH. ^**^Extra 2 pups were used for IF (Ki67, PCNA and GFAP) at the 72 h after GMH

Male and female SD rat pups were randomized into experimental and sham groups. The animal groups and experimental design are shown in Figure 1. Each animal group had zero mortalities during these experiments.

Secukinumab (TM‐Secu‐00002‐6; TheraMabs Bio‐Technology Inc) at dosages of 0.3, 0.6 and 0.9 mg/kg for best dose‐response or phosphate‐buffered saline (PBS, sham and vehicle) was intranasally administered at 1 hour postictus. Dose concentrations for secukinumab were adapted from the following manuscript.^32^ In some treatment groups, two interventions were used to delineate the downstream signaling pathway. C/EBPβ CRISPR activation plasmid (2 μg/pup, sc‐437357‐ACT, Santa Cruz Biotechnology, Inc) or its solvents (vehicle) were administered by intracerebroventricular injection (icv) 48 hours before GMH induction. SIRT1 antagonist EX527 (5 μg/pup, ab141506, Abcam) or DMSO (vehicle) was delivered by icv 1 hour before GMH induction.

### 2.3 Neurobehavioral assessments

Short‐term behavior (24, 48 and 72 hours postictus) was evaluated using righting reflex and negative geotaxis (at 90° and 180° inclined surface) as previously described.^33^ Morris water maze was used to evaluate spatial learning and memory at 24‐28 days postictus, and motor function was assessed by foot fault test and Rotarod at 28 days postictus as previously described.^33^ Neurobehavioral function was evaluated in a blinded manner.

### 2.4 Animal perfusion and tissue extraction

Animals were euthanized using isoflurane (≥5%) which was followed by transcardiac perfusion with ice‐cold phosphate‐buffered saline (PBS) for Western blot or ice‐cold PBS followed by 10% formalin for histology samples.

### 2.5 Western blotting

Ipsilateral forebrain samples were extracted from experimental rat pups for Western blot (WB) samples as previously described.^34^ Samples were homogenized in RIPA lysis buffer (sc‐24948, Santa Cruz, USA) followed by centrifugation at 14 000 for 30 minutes; once finished, the supernatant was removed from the remainder of the sample. Protein concentrations for immunoblotting were determined by detergent‐compatible protein assay (Bio‐Rad). 5 uL (1.2 mg/mL) protein per sample was loaded into each well located on 7.5%‐15% SDS‐PAGE. First, gels ran for 30 minutes at 50 V, which was followed by 90 minutes at 125 V. The gel would then be transferred onto nitrocellulose membranes at 0.3 A for 120 minutes (Bio‐Rad). Membranes were incubated with primary antibody at 4°C temperature overnight. Primary antibodies included used are as follows: rabbit polyclonal anti‐IL‐17A (1:500, GTX32674; Gene Tex), rabbit polyclonal anti‐IL‐17RA antibody (1:500, ab180904; Abcam), mouse monoclonal anti‐C/EBPβ antibody (1:500, ab15049; Abcam), mouse monoclonal anti‐SIRT1 antibody (1:1000, ab110304; Abcam), rabbit monoclonal anti‐CyclinD1 antibody (1:100, ab16663; Abcam), goat polyclonal anti‐GFAP antibody (1:3000, ab53554; Abcam), rabbit monoclonal anti‐PCNA antibody (1:1000, ab92552; Abcam), and mouse monoclonal anti‐actin antibody (1:5000, ab8226; Abcam). The membranes were incubated with their corresponding secondary antibodies for 2 hours at room temperature: anti‐mouse (1:3000, SC‐516102, Santa Cruz, USA); anti‐rabbit (1:3000, Santa Cruz, USA); and anti‐goat (1:5000, Santa Cruz, USA). The membrane was then exposed to radiography films to display the protein bands. Lastly, the density of bands was analyzed for the relative density of the resultant protein immunoblot by ImageJ software (NIH).^35^

### 2.6 Immunofluorescence staining

At 72 hours and 7‐days postictus, brain samples were sectioned into 10‐μm slices using a cryostat (LEICA CM 1860, Leica Microsystems). Immunofluorescence staining was performed as previously described.^34^ Sections were stained with primary antibodies of IL‐17RA (1:100, Abcam), GFAP (1:500, Abcam), NeuN (1:200, Abcam), Iba1 (1:200, Abcam), Ki67 (1:200, Abcam), or PCNA (1:100, Abcam) at 4°C overnight. They then were incubated with the appropriate fluorescence‐conjugated secondary antibodies (1:200, Jackson ImmunoResearch Labs) for 1 hour at room temperature. The perihemorrhagic area was imaged by a DMi8 fluorescent microscope (Leica Microsystems). Astrocyte scar tissue formation was observed by GFAP immunofluorescence staining; then, ImageJ software (NIH) was used to count the average density of GFAP from 4 points selected randomly from the surrounding area of the perihematoma region under a 400 × fold field.

### 2.7 Nissl staining

Nissl staining was performed as previously described in Ref.^36^ to examine the relative cortical thickness and ventricular volume.^36^, ^37^

### 2.8 Statistical analysis

Data are expressed as mean ± standard deviation and were analyzed by GraphPad Prim 7.0 software (GraphPad Software Inc). One‐way ANOVA on ranks was performed to compare the difference among each group and followed by the Tukey multiple comparison post hoc test. *P*‐value < 0.05 was considered statistically significant.

## 3 RESULTS

### 3.1 Time course study: IL‐17A, IL‐17RA, SIRT1, CyclinD1, and GFAP expression after GMH

**Figure 2:**
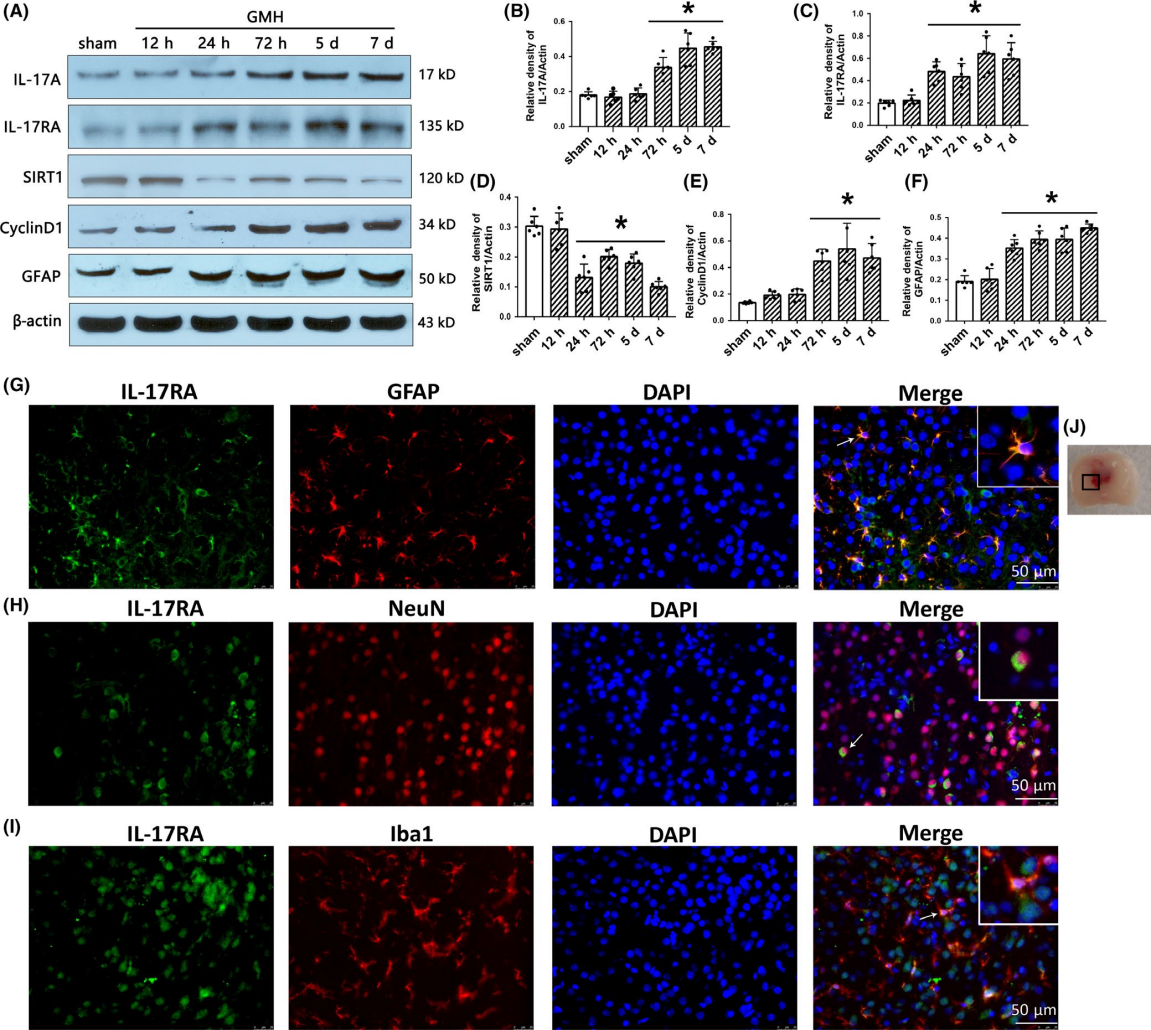
The time course of IL‐17A, IL‐17RA, SIRT1, CyclinD1, GFAP, and cellular localization of IL‐17RA after GMH. A‐F, The expression of IL‐17A, IL‐17RA, SIRT1, CyclinD1, and GFAP at different times after GMH. ^*^ *P* < 0.05 vs sham, mean ± SD, one‐way ANOVA, Tukey's test, n = 6/group. G‐I, Colocalization of IL‐17RA with GFAP, NeuN, and Iba1 at 72 h after GMH. J, indicates the photographed location. Scale bar = 50 µm, n = 2/group. GMH, germinal matrix hemorrhage. GFAP, glial fibrillary acidic protein; NeuN, hexaribonucleotide binding protein 3; Iba‐1, ionized calcium binding adapter molecule 1; DAPI, 4′,6‐diamidino‐2‐phenylindole; IL‐17RA, IL‐17A receptor

The endogenous protein levels were evaluated by Western blot at 0, 12, 24, 72 hours, and 5 and 7 days post‐GMH. Endogenous IL‐17A expression was significantly increased at 72 hours, and 5 and 7 days after GMH (Figure 2A,B). Endogenous IL‐17RA was significantly increased at 24 hours and peaked at 5 days post‐GMH (Figure 2A,C). The endogenous expression of SIRT1 was significantly decreased at 24 hours after GMH (Figure 2A,D), and proliferation marker CyclinD1 expression was significantly increased at 72 hours after GMH (Figure 2A,E). GFAP significantly increased at 24 hours after GMH and remained elevated (Figure 2A,F). Based on these results, the 5‐day time point was chosen to study the mechanism of action.

Immunofluorescence staining was used to determine the cellular localization of IL‐17RA in the CNS at 72 hours after GMH. IL‐17RA was found to be colocalized with GFAP, NeuN, and Iba1 at the site of perihematoma (Figure 2G‐J). These results indicate that IL‐17RA is expressed on astrocyte, neuronal, and microglia cells.

### 3.2 Secukinumab improved short‐ and long‐term neurological function after GMH

**Figure 3:**
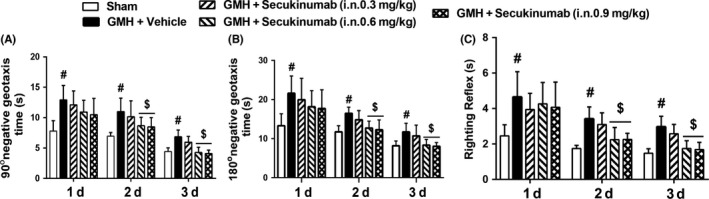
Intranasal administration of secukinumab improved short‐term neurological function in the 2 and 3 d after GMH. A, 90° negative geotaxis; B, 180° negative geotaxis; C, righting reflex. ^#^ *P* < 0.05 vs sham, ^$^ *P* < 0.05 vs GMH+ vehicle, mean ± SD, one‐way ANOVA, Tukey's test, n = 6/group. GMH, germinal matrix hemorrhage

Short‐term neurological function was examined in the following animal groups for the best dose‐response study: sham, GMH + vehicle, GMH + secukinumab 0.3 mg/kg, GMH + secukinumab 0.6 mg/kg, and GMH + secukinumab 0.9 mg/kg animal groups. GMH rats had significantly short‐term neurological deficits compared to shams. At 24 hours, all groups had significant neurological impairment in all neurobehavioral tests when compared to sham (Figure 3A,B). However, secukinumab at dosages of 0.6 mg and 0.9 mg/kg significantly improved short‐term neurological outcomes at the 2 and 3 days after GMH (*P* < 0.05, Figure 3A,B). Based on the short‐term neurological function outcomes, 0.6 mg/kg was chosen as the best dose and is used for the following experiments.

**Figure 4:**
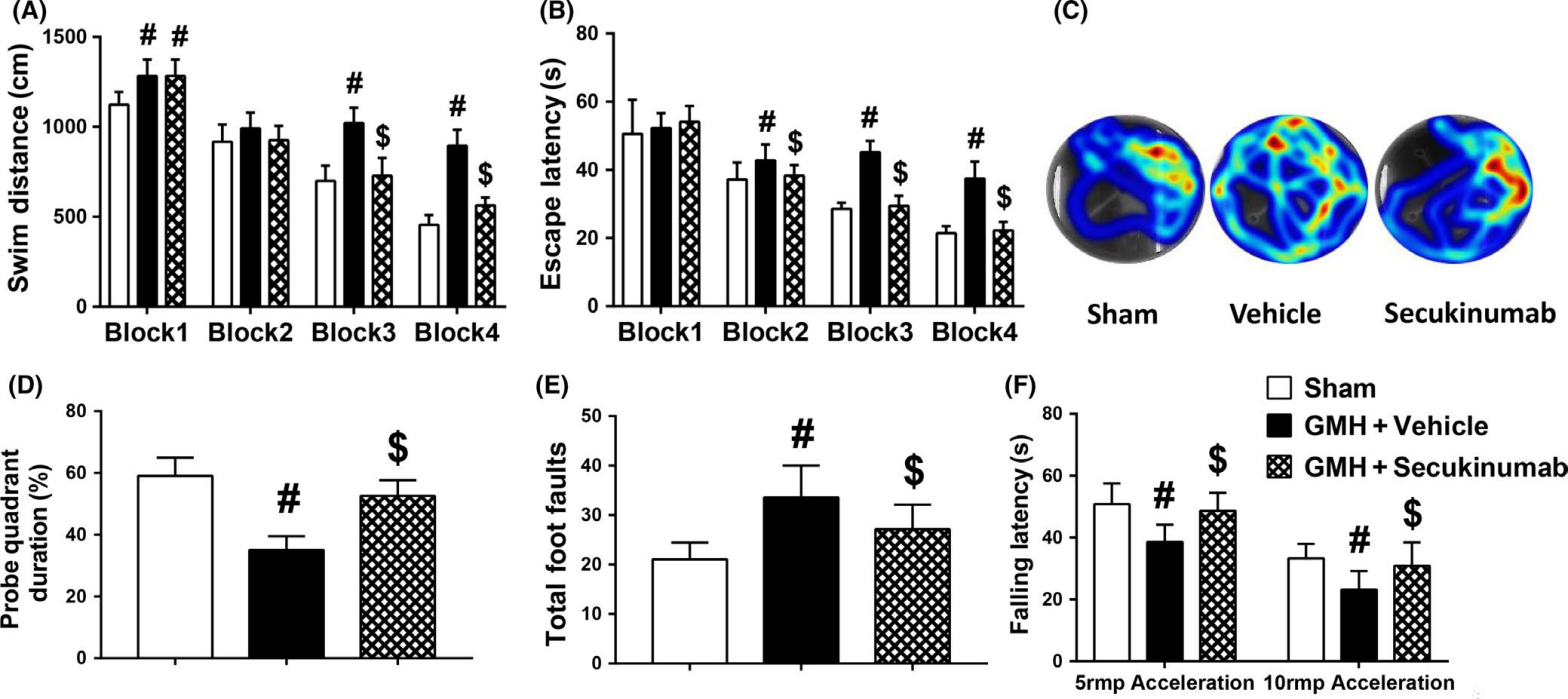
Secukinumab (0.6 mg/kg, in) treatment improved memory and motor function at 24‐28 d after GMH. A‐D, Water maze tests, (E) Rotarod tests and (F) foot fault tests. ^#^ *P* < 0.05 vs sham, ^$^ *P* < 0.05 vs vehicle, mean ± SD, one‐way ANOVA, Tukey's test, n = 10/group. GMH, germinal matrix hemorrhage

For seven consecutive days, Morris water maze was performed to evaluate spatial learning and memory starting at 24 days after GMH. The vehicle group performed significantly worse on all days compared to sham, whereas secukinumab treatment significantly improved spatial learning and memory in which this animal group's (a) swim distance in block 3 and block 4 (Figure 4A), and (b) escape latency from block 2 to block 4 (Figure 4B) were significantly decreased when compared to vehicle. Additionally, the percentage of time spent in the probe quadrant was significantly higher in the treatment group when compared to the vehicle group (Figure 4C,D). At 28 days after GMH, sensorimotor function was significantly improved in the secukinumab‐treated group when compared to vehicle (Figure 4E,F).

### 3.3 Secukinumab reduced ventricular volume and preserved cortical thickness after GMH

**Figure 5:**
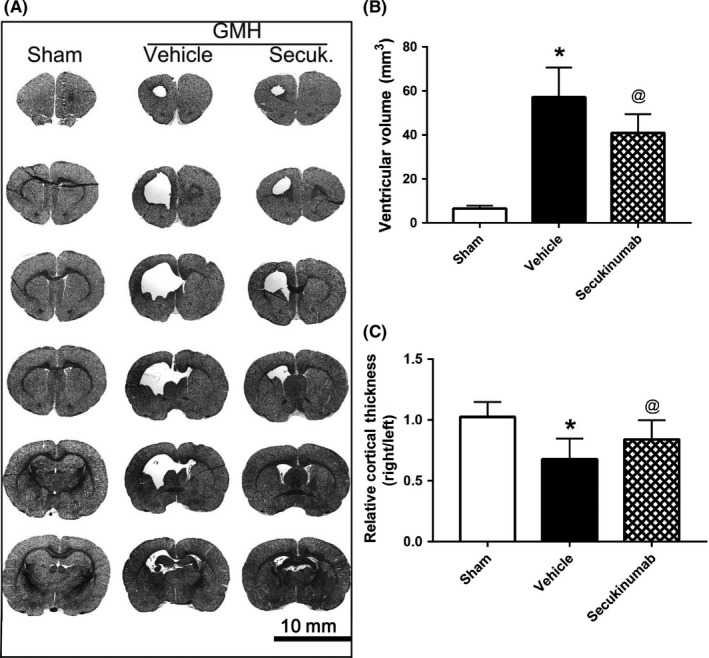
Secukinumab (0.6 mg/kg, in) treatment reduced the ventricular volume and increased the cortical thickness of ipsilateral brain 28 d after GMH. A, Representative Nissl staining images; B, ventricular volume; C, the relative cortical thickness. ^*^ *P* < 0.05 vs Sham, ^@^ *P* < 0.05 vs vehicle, mean ± SD, one‐way ANOVA, Tukey's test, n = 10/group. GMH, germinal matrix hemorrhage

Nissl staining was performed to evaluate ventricular volume and relative cortical thickness at 28 days after GMH. Compared with sham, the vehicle group had significantly enlarged ventricles and decreased cortical thickness, while secukinumab‐treated group significantly attenuated ventricular dilation and preserved cortical thickness (Figure 5).

### 3.4 Secukinumab reduced astrogliosis around the site perihematoma after GMH

**Figure 6:**
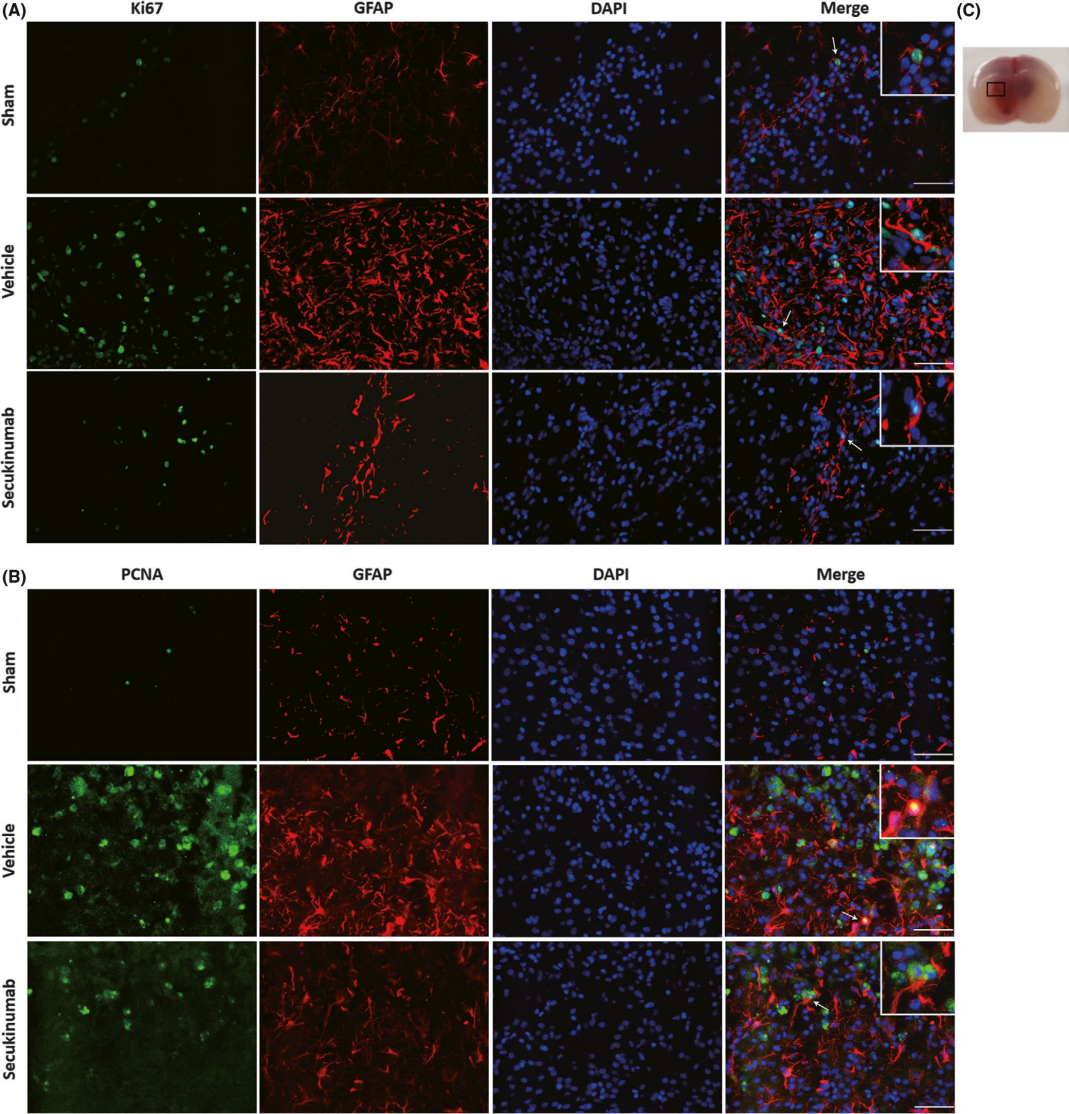
Secukinumab (0.6 mg/kg, in) treatment decreased astrocyte proliferation at 72 h after GMH in rat pups. A, Colocalization of Ki67 with GFAP. B, Colocalization of PCNA with GFAP. C, indicates the photographed location. Scale bar = 50 µm, n = 2/group. DAPI, 4′,6‐diamidino‐2‐phenylindole; GFAP, glial fibrillary acidic protein; GMH, germinal matrix hemorrhage; PCNA, proliferating cell nuclear antigen

At 72 hours postictus, astrocyte proliferation was accessed by immunofluorescence to determine the effect of the treatment. Proliferation markers Ki67 or PCNA were colocalized with astrocyte marker GFAP. Ki67‐ or PCNA‐positive astrocyte expression was increased in the vehicle group when compared to shams. Secukinumab treatment reduced Ki67‐ or PCNA‐positive astrocyte expression (Figure 6A,B).

**Figure 7:**
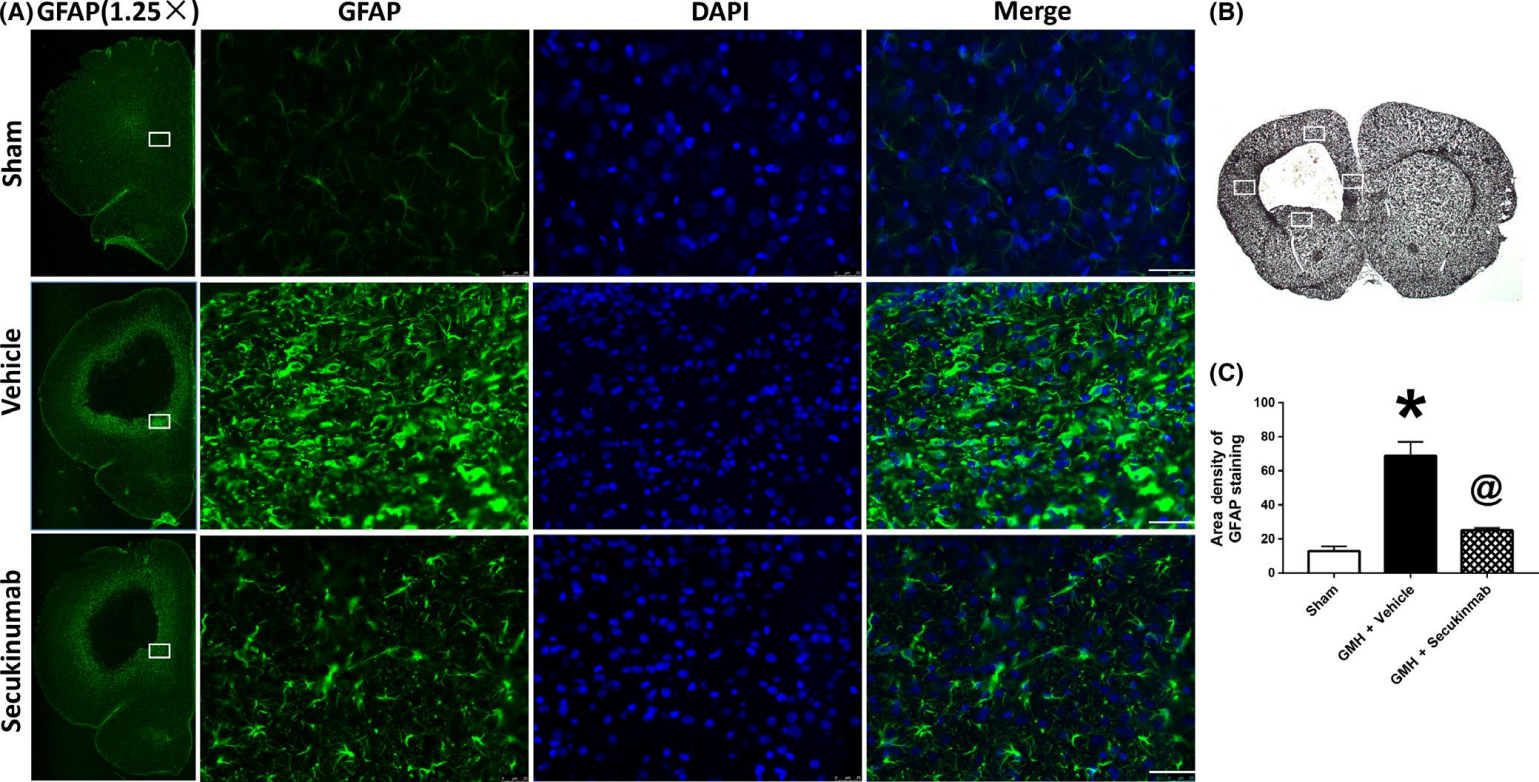
Secukinumab reduced astrocyte scar formation at 7 d after GMH. A, Repetitive images of GFAP staining. B, indicates the photographed areas. C, Quantitative analysis of area density of GFAP staining. Scale bar = 50 µm, ^*^ *P* < 0.05 vs sham, ^@^ *P* < 0.05 vs GMH+ vehicle, mean ± SD, one‐way ANOVA, Tukey's test, n = 6/group. DAPI, 4′,6‐diamidino‐2‐phenylindole; GFAP, glial fibrillary acidic protein; GMH, germinal matrix hemorrhage

Immunohistochemical staining for GFAP at 7 days postictus indicated an increase in glial scar formation around the site of perihematoma, and secukinumab treatment significantly decreased the density of scarring around this area (Figure 7A,C).

### 3.5 The SIRT1 inhibitor EX527 or C/EBPβ CRISPR activation plasmid reversed the beneficial effects of secukinumab after GMH

**Figure 8:**
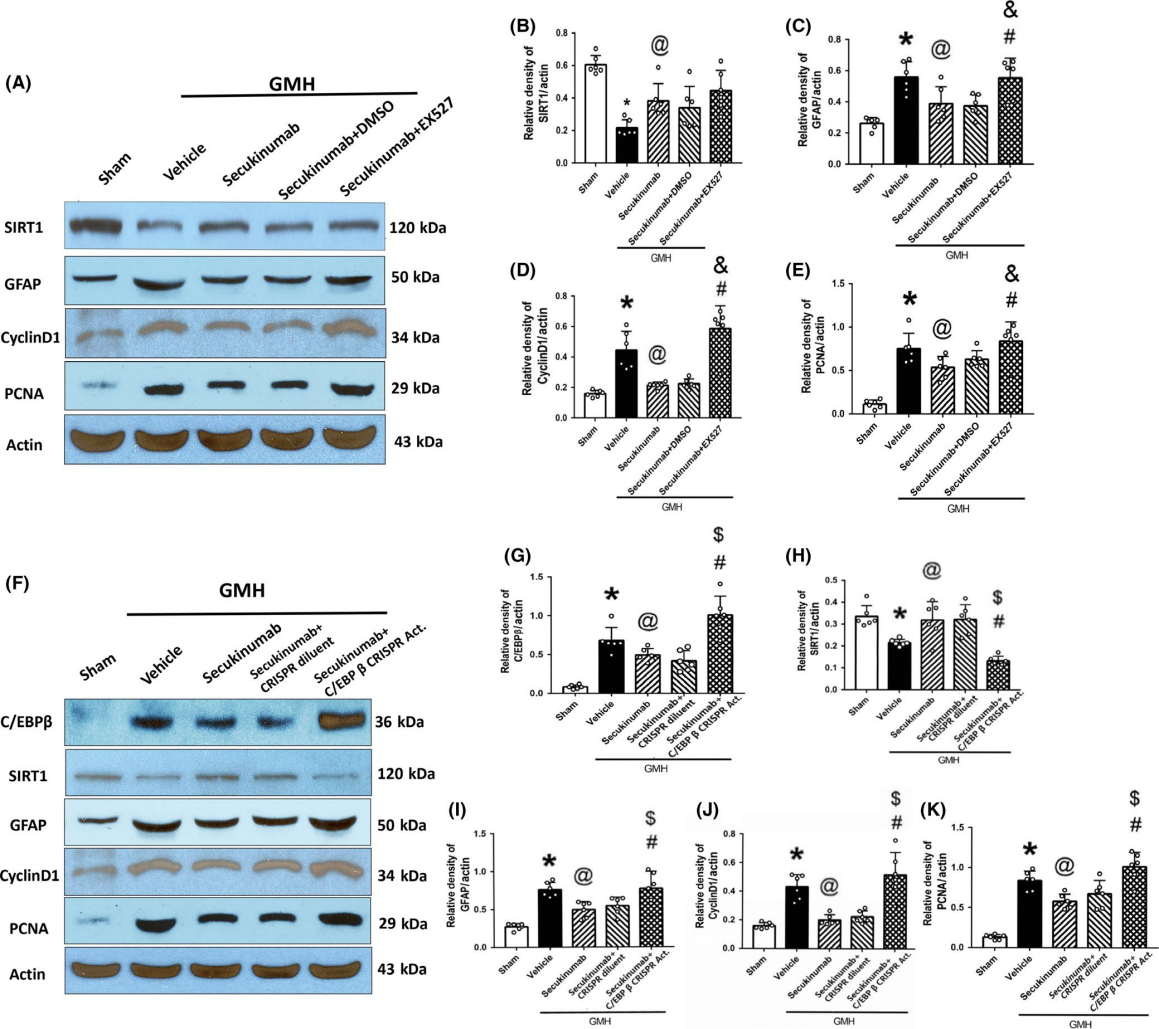
The administration of EX527 and C/EBPβ CRISPR reversed the antireactive astrogliosis effect of secukinumab. A‐E, The representative bands and quantitative analysis of SIRT1, GFAP, CyclinD1, PCNA intervened with EX527; F‐K, The representative bands and quantitative analysis of C/EBPβ, SIRT1, GFAP, CyclinD1, and PCNA intervened with C/EBPβ CRISPR activation plasmid. ^*^ *P* < 0.05 vs Sham, ^@^ *P* < 0.05 vs vehicle, ^&^ *P* < 0.05 vs secukinumab + DMSO, ^#^ *P* < 0.05 vs secukinumab, ^$^ *P* < 0.05 vs secukinumab + CRISPR diluent, ^$^ *P* < 0.05 vs secukinumab, mean ± SD, one‐way ANOVA, Tukey's test, n = 6/group. C/EBPβ, CCAAT/enhancer binding proteins beta; CRISPR, clustered regularly interspaced short palindromic repeats; DMSO, dimethyl sulfoxide; GFAP, glial fibrillary acidic protein; GMH, germinal matrix hemorrhage; PCNA, proliferating cell nuclear antigen; SIRT1, silent information regulator 1

IL‐17 activation of C/EBPβ and its inhibition of SIRT1 play a role in astrocyte proliferation that leads to glial scar formation after GMH. Thus, we evaluated the effects of the treatments on SIRT1, CyclinD1, PCNA, and C/EBPβ at 5 days postictus. The vehicle group significantly increased C/EBPβ expression, proliferation markers (CyclinD1 and PCNA), and GFAP, and decreased expression of SIRT1 (Figure 8A‐E). Secukinumab treatment significantly decreased the C/EBPβ expressions, proliferation markers, and GFAP, and increased SIRT1 expression (Figure 8A‐E). SIRT1 inhibitor EX527 and C/EBPβ CRISPR activation plasmid reversed the effects of secukinumab on (C/EBPβ), proliferation markers, GFAP, and SIRT1 (Figure 8F‐K).

## 4 DISCUSSION

In this present study, we first time investigated the therapeutic effects of secukinumab against reactive astrogliosis by inhibiting IL‐17A signaling pathway in a collagenase‐induced rat pup model of GMH. The following observations were made: (a) Endogenous protein levels of IL‐17A and its receptor IL‐17RA were significantly expressed over the course of 7 days postictus, which contributed to the endogenous reduction of SIRT1 expression and increased cell proliferation markers (CyclinD1 and PCNA). Additionally, the following was elucidated from this study: (a) IL‐17RA was shown to be expressed on astrocytes, neurons, and microglia cells after GMH; (b) secukinumab treatment, administered intranasally at 1 hour after GMH, significantly improved short‐ and long‐term neurobehavioral performance after GMH; (c) secukinumab treatment significantly reduced ventricular dilation and increased ipsilateral cortical thickness after GMH; (d) secukinumab decreased astrocyte proliferation and glial scarring near the site of perihematoma in secukinumab‐treated group; and (e) C/EBPβ CRISPR activation plasmid and EX527 inhibitor abolished the anti‐astrogliosis effects of secukinumab.

Germinal matrix hemorrhage is a devastating disorder in newborn infants.^2^ Our well‐established rat pup model of GMH, which mimics the neurological deficits in this human newborn patient population,^33^ demonstrated that reactive astrogliosis plays a vital role in this pathophysiology. Reactive astrogliosis is characterized by astrocyte proliferation, neuronal dysfunction, cellular hypertrophy, and upregulation of intermediate filament proteins such as GFAP which can lead to glial scar formation.^7^, ^38^ Reactive astrogliosis has been shown to be a byproduct of stroke, causing changes to the brain tissue, blood vessel, CNS microenvironment, and disrupting neurotransmissions.^14^ During the acute phase of CNS injury, the proliferation of astrocytes occurred which contributed to cell death, neovascular remodeling, multicellular inflammation, loss of axons and synapse in perilesion, and loss of oligodendrocytes.^38^ Astrocytes play a primary role in the production of inflammatory cytokines, chemokines, and ROS,^13^ leading to the inhibition of oligodendrocyte progenitor differentiation and hydrocephalus, resulting in secondary brain injury.^39^ Then newly proliferated and hypertrophic astrocyte cells, fibroblast‐linage cells and inflammatory cells consisted of astrogliosis scar formation under modulating by intrinsic signaling transduction such as STAT3 and NF‐ƙB pathway.^38^, ^40^, ^41^ After the acute phase of brain injury, scar formation attenuated neuronal regeneration, blocked axon prolongation, inhibited synapse remodeling, and reduced the thickness of ipsilateral cortex,^14^, ^15^ leading to long‐term neurobehavioral deficits. Additionally, glial scarring blocks the circulation of cerebrospinal fluid (CSF) and contributed to the enlargement of ventricles, which impaired neurological function.^42^ Thus, targeting reactive astrogliosis may be beneficial for the treatment of GMH.

IL‐17A, secreted by immune cells,^43^, ^44^ plays a vital role in the stroke pathophysiology. In addition to its pro‐inflammatory effect, IL‐17A has been shown to promote hyperproliferation and differentiation of various cell types; the most notable are Th17 cells and γδT cells due to their production of IL‐17A.^17^, ^45^, ^46^, ^47^, ^48^ After hemorrhagic stroke, macrophages become activated and secret IL‐23, which promotes the activation of γδT cells and Th cells.^18^ Due to the blood‐brain barrier (BBB) rupture after hemorrhagic stroke,^49^ IL‐17A enters into the brain tissue through the serum.^50^ The expression of IL‐17A has been shown to increase in both the serum and brain tissue after hemorrhagic stroke,^50^, ^51^ We observed increased IL‐17A receptor (IL‐17RA) expression on astrocytes, in concurrence to previous reports.^52^

IL‐17 receptor (IL‐17RA) and its complexes have no intracellular enzymatic activity but implement their function by protein‐protein signal transmission.^53^ The most notable pathway is Act1 (one of two IL‐17RA complexes) activation of C/EBPβ.^17^, ^53^ Various articles indicate that C/EBPβ is expressed in astrocytes and a plays role in inflammation, cell proliferation, and metabolism.^24^, ^54^ After activation, C/EBPβ is rapidly transported to the nucleus and binds to HDAC1. Consequently, the C/EBPβ‐HDAC1 complex was found to negatively regulate SIRT1 expression and activity.^24^, ^25^ SIRT1 plays a significant role in the deacetylation of NF‐ƙB,^29^, ^55^ which resulted in a decrease in proliferation proteins such as CyclinD1 and PCNA.^29^, ^56^ Additionally, SIRT1 has been shown to downregulate STAT3^30^ and reduced reactive astrogliosis.^57^ In this study, we observed a significant reduction in endogenous SIRT1 expression along with an increase in cell proliferation marker CyclinD1 after GMH. The time course of these markers showed a similar trend to that of IL‐17A and IL‐17AR, suggesting that IL‐17A/IL‐17AR/SIRT1 signaling pathway may play a role in the regulation of reactive astrogliosis after GMH. We also observed that secukinumab, which selectively targets IL‐17A,^32^ significantly improved short‐ and long‐term neurological deficits induced by GMH. These behavioral benefits were associated with reduced ventricular dilation, decreased astrocyte activation and proliferation, and reduced glial scar tissue formation around the site of perihematoma.

To study the secukinumab mechanism of action, we evaluated its effects on the (C/EBPβ)/SIRT1 axis. C/EBPβ CRISPR activation plasmid ameliorated the therapeutic effects of secukinumab on astrocyte proliferation and activation, which was associated with increased expression of C/EBPβ, CyclinD1, PCAN, and GFAP, and decreased SIRT1 expression. SIRT1 inhibitor EX527 also reversed the effect of secukinumab. This study demonstrated that secukinumab attenuated reactive astrogliosis after GMH through the attenuation of the IL‐17RA/(C/EBPβ) signaling pathway, removing the inhibitory effects on SIRT1.

There are some limitations to our study. First, reactive astrogliosis is defined as changes in cell morphology, proliferation, function, and gene expression.^7^, ^38^ However, we primarily evaluated the effect of secukinumab on astrocyte morphology and proliferation. Second, we did not measure the concentration of secukinumab in serum or brain tissue. Third, IL‐17RA was also expressed in neurons and microglia. Secukinumab effects on these cell types need further investigation. Lastly, secukinumab and SIRT1 have been shown to be anti‐inflammatory in several CNS injury models.^58^, ^59^ Because of this, secukinumab's reduction of reactive astrogliosis cannot be entirely attributed as the sole cause of neuroprotection in GMH‐treated animals.

## 5 CONCLUSION

In the current study, we demonstrated that secukinumab treatment attenuated neurological deficits and reactive astrogliosis after GMH in rat pups. The protective effects were mediated by the inhibition of IL‐17RA/(C/EBPβ) signaling pathway. Our study is the first to demonstrate secukinumab effects on glial scarring, providing new insight for therapeutic strategies for the management of patients with GMH.

## CONFLICT OF INTEREST

The authors declare no conflicts of interest.
